# TRIM32 promotes cell proliferation and invasion by activating β‐catenin signalling in gastric cancer

**DOI:** 10.1111/jcmm.13784

**Published:** 2018-08-05

**Authors:** Changming Wang, Jiapeng Xu, Hongbing Fu, Yu Zhang, Xin Zhang, Dejun Yang, Zhenxin Zhu, Ziran Wei, Zunqi Hu, Ronglin Yan, Qingping Cai

**Affiliations:** ^1^ General Surgery Department Shanghai Baoshan Hospital of Integrated Traditional Chinese Medicine and Western Medicine Shanghai University of Traditional Chinese Medicine Shanghai 201999 China; ^2^ Department of Gastrointestinal Surgery Changzheng Hospital Second Military Medical University Shanghai China

**Keywords:** β‐catenin, gastric cancer, invasion, proliferation, TRIM32

## Abstract

The tripartite motif (TRIM) family comprises more than 70 members involved in the regulation of many cellular pathways. TRIM32 acts as an E3 ubiquitin ligase and has been reported to participate in many human cancers. Here, we aimed to investigate the role of TRIM32 in gastric cancer (GC) and the clinical implications. High expression of TRIM32 was observed in GC tissues and cell lines, and was significantly associated with poor prognosis. Knockdown TRIM32 expression remarkably suppressed the proliferation, migration, and invasion of GC cells in vitro and tumour growth in vivo, whereas overexpression of TRIM32 yielded the opposite results. Western blotting and quantitative reverse‐transcription PCR (qRT‐PCR) analyses revealed that up‐regulation of TRIM32 significantly enhanced expression of β‐catenin protein and of its downstream targets TCF1, cyclin D1, Axin2 and MMP7 mRNAs. Moreover, we found that the mechanism behind the TRIM32‐promoted GC progression was related to the β‐catenin signalling pathway. Collectively, these data suggest that TRIM32 promotes GC cell proliferation, migration, and invasion by activating the β‐catenin signalling pathway.

## 1 INTRODUCTION

Gastric cancer (GC) is the fourth most frequent malignant tumour and the second leading cause of cancer‐related deaths worldwide.^1^ GC is an aggressive tumour that is usually diagnosed at a late stage. Although significant progress has been made in treatment, GC remains a serious health problem with a low 5‐year survival rate.^2^ Therefore, an increasing number of studies have focused on the molecular mechanisms underlying gastric carcinogenesis, and these data may help to identify novel therapeutic targets.

Proteins of the tripartite motif (TRIM) family are characterized by the presence of an N‐terminal RING finger followed by 1 or 2 B‐boxes (type 1 and type 2) and a coiled‐coil region.^3^ Most of TRIM family members act as E3 ubiquitin ligases that promote post‐translational modifications of various substrates.^4^ TRIM family proteins participate in a wide range of biological processes, such as cell growth, migration, differentiation, apoptosis, and immunity.^5^, ^6^, ^7^, ^8^ In the past few years, the role of TRIM proteins received much attention regarding innate immunity to viral infections.^9^ Recently, researchers found that TRIM proteins also serve as oncogenes or tumour suppressors implicated in various cancers, including GC. For example, TRIM25 blockade by RNA interference inhibits migration and invasiveness of GC cells through TGF‐β signalling.^10^ Increased TRIM29 mRNA expression is markedly associated with histological grade, large tumour size, the extent of tumour invasion, and lymph node metastasis and performs a function of an oncogene in GC.^11^, ^12^ TRIM44 plays a crucial role in tumour cell proliferation through its overexpression, and research has highlighted its usefulness as a predictor and potential therapeutic target in GC.^13^ Whether TRIM32—as an evolutionarily conserved TRIM family member—also contributes to GC pathogenesis needs to be explored.

In this study, we showed that TRIM32 is up‐regulated in GC tissues and cell lines and is associated with an unfavourable prognosis. TRIM32 promotes GC cell proliferation, migration, invasion, and xenograft tumour growth. Mechanistically, forced expression of TRIM32 in GC cells correlated with up‐regulation of β‐catenin, whereas inhibition of TRIM32 reduced β‐catenin expression. Thus, TRIM32 helps to promote gastric carcinogenesis by up‐regulating the β‐catenin pathway.

## 2 MATERIALS AND METHODS

### 2.1 Patients’ samples and cell lines

Paraffin‐embedded tissues of a training group of 61 patients with GC were collected at Changzheng Hospital (Shanghai, China). Another 20 fresh GC samples and adjacent non‐cancerous tissues were also obtained from the hospital. None of the patients received chemotherapy or radiotherapy before gastrectomy. The fresh samples were frozen in liquid nitrogen and stored at −80°C for the subsequent experiments. This study's protocol was approved by the Ethics Committee of Changzheng Hospital. All the patients provided written informed consent. Human GC cell lines SGC7901, BGC823, AGS and MKN28 and immortalized gastric epithelial cell line GES‐1 were obtained from the Cell Resource Center of the Chinese Academy of Sciences (Shanghai, China). Cells were maintained in the RPMI 1640 medium (Life Technologies, Grand Island, NY, USA), supplemented with 10% of foetal bovine serum (FBS; HyClone, Logan, UT, USA) and cultured in a humidified chamber at 5% CO_2_ and 37°C.

### 2.2 Vector construction and cell transfection

The coding sequences of TRIM32 were cloned into the pCDH‐CMV‐MCS‐EF1‐coGFP vector (System Biosciences, Mountain View, CA, USA). TRIM32 short hairpin RNA (shRNA) (target sequence: 5′‐GGUGGAAAGCUUUGGUGUU‐3′) was cloned into the pLKO.1 vector. Lentiviral particle production was performed according to other reports.^14^ Cells were infected with recombinant lentivirus‐transducing units plus 5 mg/mL Polybrene (Sigma, St Louis, MO, USA).

### 2.3 RNA extraction and quantitative reverse‐transcription PCR (qRT‐PCR)

Total RNA was isolated from tissue samples and from cell lines using the TRIzol Reagent (Life Technologies). The quality of the RNA was measured on a NanoDrop ND‐1000 spectrophotometer (NanoDrop Technologies, Wilmington, DE, USA). Total RNA and the PrimeScript RT Reagent (Takara, Dalian, China) were used to generate cDNA, which was subjected to qRT‐PCR with the SYBR Green Master Mix (Takara) performed on an ABI 7900HT Fast Real‐Time PCR System (Applied Biosystems). β‐Actin served as an endogenous control to normalize mRNA expression data. The 2^−ΔΔct^ method was applied to quantify the mRNA. Each experiment was conducted in triplicate.

### 2.4 Western blotting

The proteins were separated by standard SDS‐PAGE in a 10% gel followed by transferring the proteins to polyvinylidene difluoride (PVDF) membranes, which were then incubated in a blocking solution (5% non‐fat dried milk). The proteins were reacted with primary antibodies at 4°C overnight and incubated with a secondary antibody at room temperature for 30 minutes. Staining was detected by enhanced chemiluminescence. The antibody against TRIM32 was purchased from Proteintech (Wuhan, China). The antibodies against TCF1, cyclin D1, β‐catenin, lamin B and GAPDH were acquired from Cell Signaling Technology (Beverly, MA, USA).

### 2.5 CCK‐8 assay

Cell Counting Kit 8 (CCK‐8) was employed to determine the cell viability of infected cells (3000 cells/well), which were seeded in 96‐well plates in RPMI‐1640. After 24, 48, 72, and 96 hours, 10 μL of CCK‐8 was added into each well and incubated for 1 hour. Absorbance at 450 nm was detected on a microplate reader (Bio‐Rad, Hercules, CA, USA). Data were calculated from three independent experiments, and each was conducted in triplicate.

### 2.6 Colony formation assay

The colony formation ability of GC cells was determined by this assay. In particular, 500 cells per well were seeded in six‐well plates and cultured with RPMI 1640 containing 10% of FBS for 2 weeks. Colonies were fixed with methanol and stained with 0.1% crystal violet (1 mg/mL).

### 2.7 Transwell migration and invasion assays

For the migration assay, the cells were plated in the upper chamber in the serum‐free medium. The medium containing 20% of FBS in the lower chamber served as a chemoattractant. After incubation for 24 hours, cells in the upper chambers were removed, and cells that migrated to the lower surface of filter were fixed and stained. For the invasion assay, cells were added into the upper chamber of Matrigel‐coated inserts in 24‐well plates and incubated for 24 hours with the serum‐free medium. Non‐invading cells were removed; cells attached to the bottom of the membrane were fixed, stained, and counted under an inverted microscope.

### 2.8 The mouse xenograft model of GC

Cells (2 × 10^6^) infected with the shTRIM32 lentivirus or TRIM32 overexpression lentivirus were implanted into the left flanks of BALB/c nude mice followed by regular monitoring of tumour growth every 4 days. After 28 days, the mice were killed, and the explants were excised and images were captured. The mice were purchased from the Shanghai Experimental Animal Center of the Chinese Academy of Sciences. The animal experiment was carried out in accordance with the National Institute of Health Guide for the Care and Use of Laboratory Animals, with the approval of the Scientific Investigation Board of the Second Military Medical University (Shanghai, China).

### 2.9 Immunohistochemistry (IHC)

Immunohistochemistry was performed by means of a Dako Envision System (Dako, Carpinteria, CA, USA).^15^ The TRIM32 antibody (Proteintech Group) served as the primary antibody. Scoring was conducted as described previously.^16^ The proportion of the immunopositive area was scored (0, 0% cells; 1, <5% cells; 2, 5%‐50% cells; 3, >50% cells), and the intensity of expression was determined (0, none; 1, low; 2, intermediate; 3, strong). The score of TRIM32 was the product of proportion and intensity scores, ranging from 0 to 6. The median IHC score was chosen as the cut‐off value for defining high and low expression.

### 2.10 Statistical analysis

Data were expressed as mean ± SD. Statistical analysis was performed in the GraphPad Prism 5.0 software. Student's *t* test was carried out for comparing two groups, whereas one‐way ANOVA was conducted for three or more groups. The Kaplan‐Meier test was used for the univariate survival analysis. All the experiments were independently conducted 3 times. Data with *P *<* *0.05 were considered statistically significant.

## 3 RESULTS

### 3.1 Increased expression of TRIM32 correlates with poor prognosis in GC

**Figure 1:**
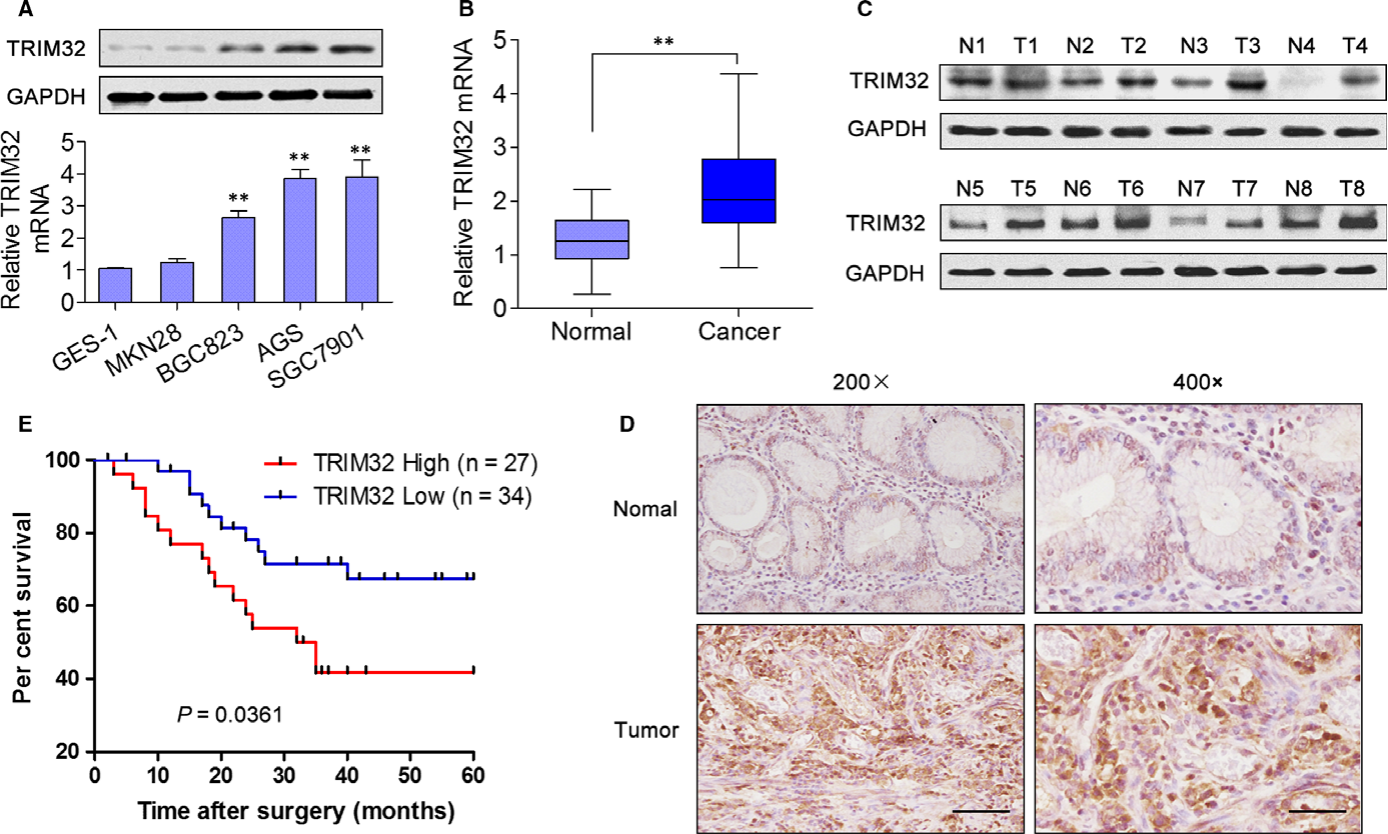
TRIM32 Expression is Increased in gastric cancer (GC) and Associated with the Prognosis. A, The mRNA and protein expression of TRIM32 was examined by qRT‐PCR and Western blotting in GES‐1 cells and 4 GC cell lines. B, The mRNA expression of TRIM32 in 20 pairs of GC tissue samples and adjacent non‐cancerous tissues. C, The protein expression of TRIM32 in 8 pairs of GC tissues and adjacent non‐cancerous tissues. D, IHC staining of 61 GC tissues and adjacent non‐cancerous tissues from GC patients. Left (200×) scale bars: 100 μm, right (400×) scale bars: 50 μm. E, Kaplan‐Meier analyses were carried out to reveal the relevance of TRIM32 expression levels to overall survival. **P <* *0.01. Data represent the mean ± SD

We first examined mRNA and protein levels of TRIM32 in four human GC cell lines (MKN28, BGC823, AGS and SGC7901) and GES‐1, an immortalized gastric epithelial cell line. qRT‐PCR and Western blotting analyses revealed that TRIM32 expression was markedly up‐regulated in 3 of the 4 GC cell lines that we studied as compared with GES‐1 cells (Figure 1A). We then determined TRIM32 expression in 20 pairs of fresh GC samples and corresponding adjacent tissue samples. The results showed that the mRNA expression of TRIM32 was significantly up‐regulated in GC samples compared to adjacent non‐cancerous tissue samples (Figure 1B). Similarly, TRIM32 protein expression was evaluated in eight pairs of GC tissues, and TRIM32 levels were found to be significantly increased in GC tissues compared to matched normal gastric tissues (Figure 1C). IHC staining confirmed that TRIM32 expression was elevated (36/61, 59%) in GC compared to matched normal gastric tissues, and the staining was mainly located in the nuclei and cytoplasm of cancer cells (Figure 1D). Moreover, it was demonstrated that patients with high TRIM32 expression had apparently poorer overall survival (Figure 1E). Taken together, these data suggested that TRIM32 overexpression may serve as a prognostic biomarker of GC.

### 3.2 TRIM32 promotes GC cell proliferation, migration and invasion in vitro

**Figure 2:**
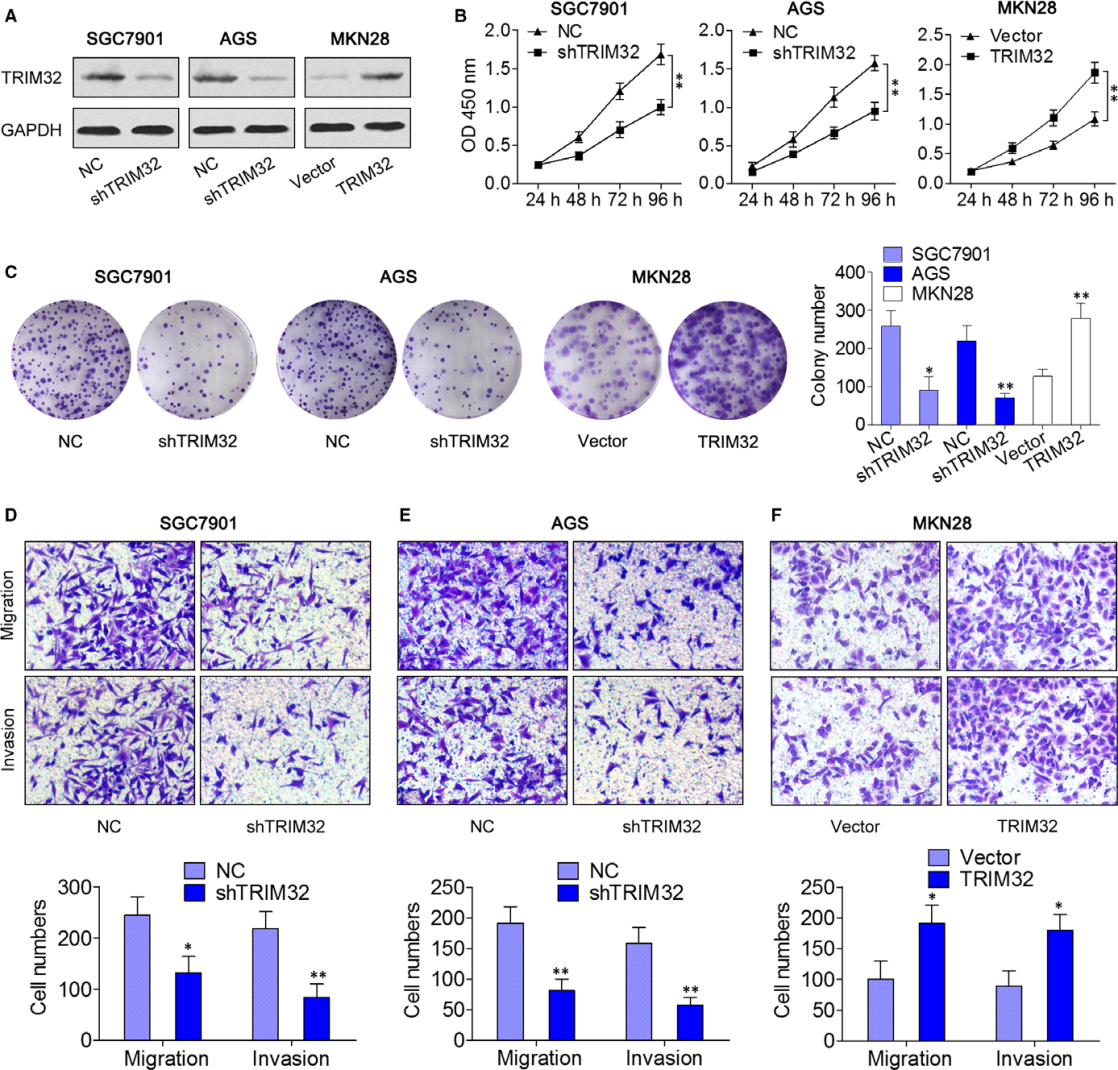
Effects of TRIM32 on the Cell Proliferation, Migration and Invasion Ability of gastric cancer (GC) Cells. A, SGC7901 and AGS cells were infected with a TRIM32 shRNA‐expressing lentivirus; MKN28 cells were infected with a lentivirus expressing TRIM32, and the expression of TRIM32 was examined by Western blotting. B, The effect of TRIM32 on cell viability was measured by the CCK‐8 assay. C, Representative pictures and quantification of colony formation assay data from shTRIM32‐ or TRIM32‐infected GC cells. D‐F, Representative images and quantification of the Transwell migration and invasion assay data from the indicated cells. *P <* *0.05, **P <* *0.01. Data represent the mean ± SD

Subsequently, we performed in vitro functional assays to explore the involvement of TRIM32 in GC progression. TRIM32 was either knocked down in SGC7901 and AGS cells or overexpressed in MKN28 cells through lentivirus transduction (Figure 2A). Results from CCK‐8 and colony formation assays indicated that TRIM32 knockdown significantly attenuated the cell proliferation ability, whereas its overexpression remarkably enhanced cell proliferation (Figure 2B,C), thus pointing to a growth‐promoting role of TRIM32 in vitro. Furthermore, Transwell migration and invasion assays revealed that knockdown of TRIM32 hindered the migration and invasion activities of SGC7901 and AGS cells (Figure 2D,E). On the contrary, up‐regulation of TRIM32 increased migration and invasiveness of MKN28 cells (Figure 2F). These results suggested that TRIM32 is involved in GC cell proliferation and invasion.

### 3.3 TRIM32 activates the β‐catenin signalling pathway

**Figure 3:**
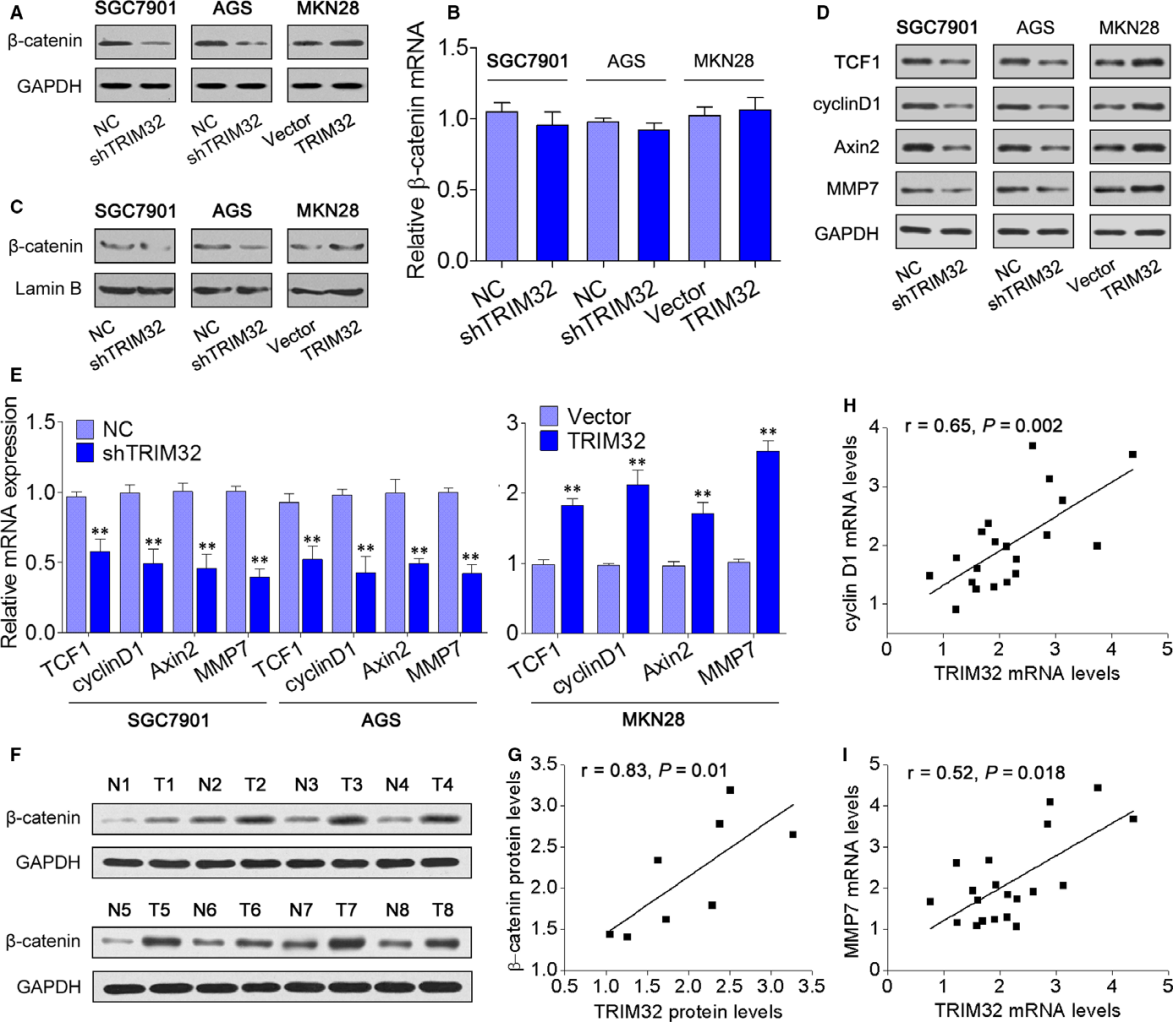
TRIM32 Activates the β‐catenin Pathway. A, Western blotting was performed to measure β‐catenin expression in SGC7901 and AGS cells expressing TRIM32 shRNA or MKN28 cells overexpressing TRIM32. B, qRT‐PCR was performed to measure β‐catenin expression in the indicated cells. C, Nuclear β‐catenin expression in the indicated cells. Lamin B served as a loading control for nuclear fraction. D,E, Expression levels of TCF1, cyclin D1, Axin2 and MMP7 in the indicated cells. F, The protein expression of β‐catenin in 8 pairs of gastric cancer (GC) tissues and adjacent non‐cancerous tissues. G, Spearman's correlation analysis of the correlation between TRIM32 and β‐catenin protein levels in GC tissues. A statistically significant positive correlation between TRIM32 mRNA level and the expression of cyclin D1 (H) and MMP7 (I) in 20 GC tissues. **P <* *0.01. Data represent the mean ± SD

To reveal the underlying mechanism by which TRIM32 promoted GC progression, we determined whether TRIM32 participates in the activation of β‐catenin signalling pathway. Strikingly, the protein expression of β‐catenin was down‐regulated by the TRIM32 knockdown in SGC7901 and AGS cells and was enhanced in MKN28 cells overexpressing TRIM32 (Figure 3A); however, inhibition or overexpression of TRIM32 had no effect on TRIM32 mRNA (Figure 3B). In the nuclear fraction, β‐catenin expression was remarkably enhanced in cells with TRIM32 overexpression and decreased in cells with TRIM32 knockdown (Figure 3C). In addition, knockdown of TRIM32 in SGC7901 and AGS cells decreased the expression of TCF1, cyclin D1, Axin2 and MMP7: four representative downstream targets of β‐catenin (Figure 3D,E). In agreement with these findings, overexpression of TRIM32 increased TCF1, cyclin D1, Axin2 and MMP7 levels in MKN28 cells (Figure 3D,E). To further examine whether the findings above could be supported by observations in human primary tumours, the correlation between TRIM32 and β‐catenin protein expression was analysed in 8 freshly collected GC tissues, and the result showed that TRIM32 expression positively correlated with β‐catenin expression (Figure 3F,G). Furthermore, a significant positive correlation was observed between TRIM32 mRNA levels and the expression of cyclin D1 and MMP7 (Figure 3H,I). Taken together, these results indicated that TRIM32 activates the β‐catenin pathway in GC cells.

### 3.4 β‐Catenin mediates the tumour ‐promoting effects of TRIM32

**Figure 4:**
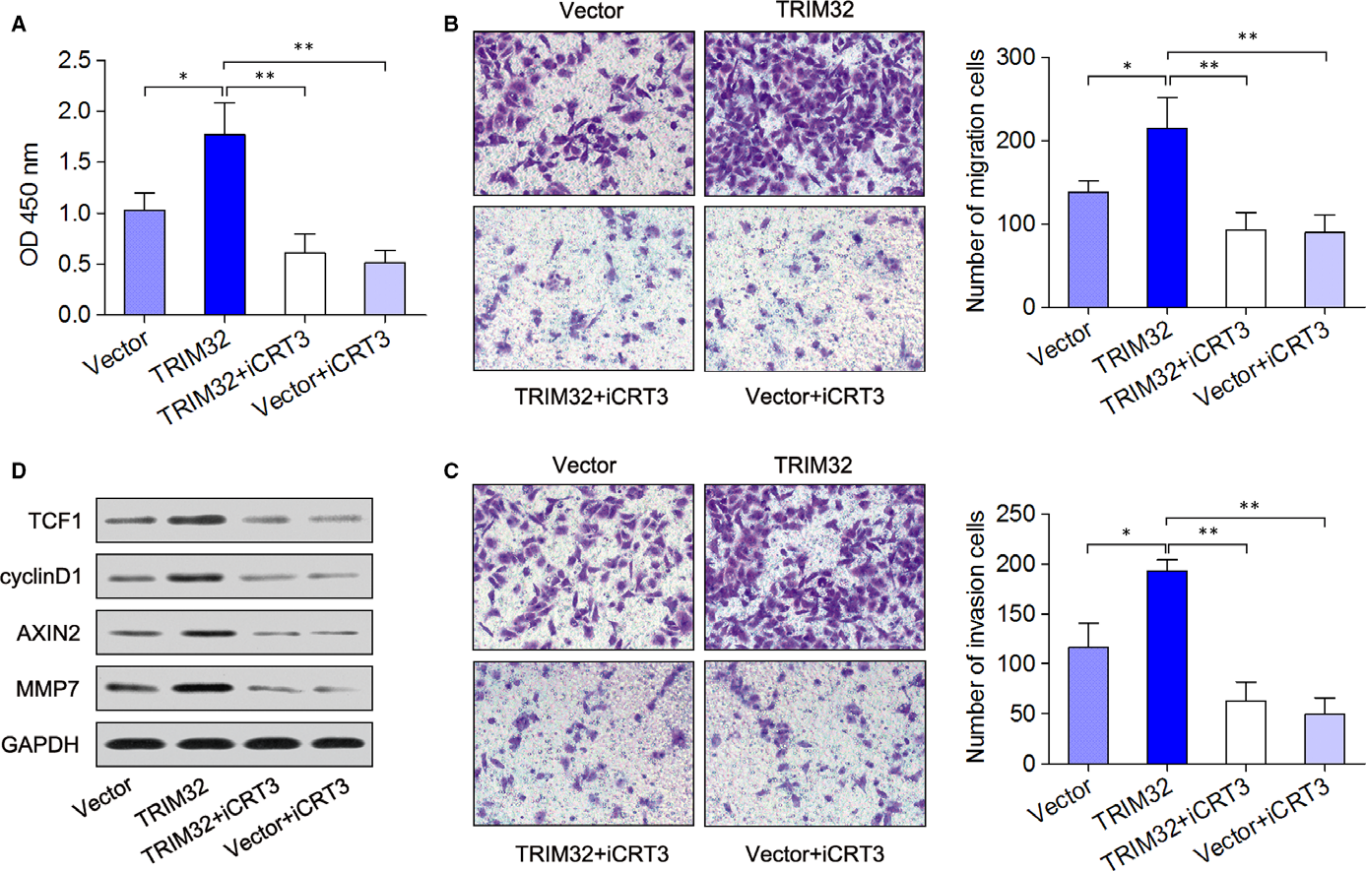
TRIM32 Exerts its Cancer‐promoting Action Through the β‐catenin Pathway. A, MKN28 cells overexpressing TRIM32 or negative control were treated with 2 μmol/L β‐catenin‐specific inhibitor iCRT3, and cell viability was determined by the CCK‐8 assay. B,C, Transwell migration and invasion assays. D, Western blotting analyses of the expression of β‐catenin, TCF1 and cyclin D1. *P <* *0.05, **P <* *0.01. Data represent the mean ± SD

To further explore the biological significance of β‐catenin in the TRIM32‐mediated tumour promotion, we studied the effect of the β‐catenin inhibitor iCRT3 on cell viability and migratory and invasive capabilities of TRIM32‐overexpressing MKN28 cells. In the CCK‐8 assay, cell viability was promoted by TRIM32 overexpression, and this increase was attenuated by the β‐catenin inhibitor iCRT3 (Figure 4B,C). Similarly, down‐regulation of β‐catenin significantly suppressed the effects of TRIM32 on cell migration and invasion (Figure 4B,C). Furthermore, overexpression of TRIM32 increased the expression of TCF1, cyclin D1, Axin2 and MMP7, and these effects were reversed after treatment with the β‐catenin inhibitor (Figure 4D). Altogether, these results suggested that the mechanism underlying TRIM32‐promoted GC progression was related to the β‐catenin signalling pathway.

### 3.5 TRIM32 promotes gastric tumour growth in vivo

**Figure 5:**
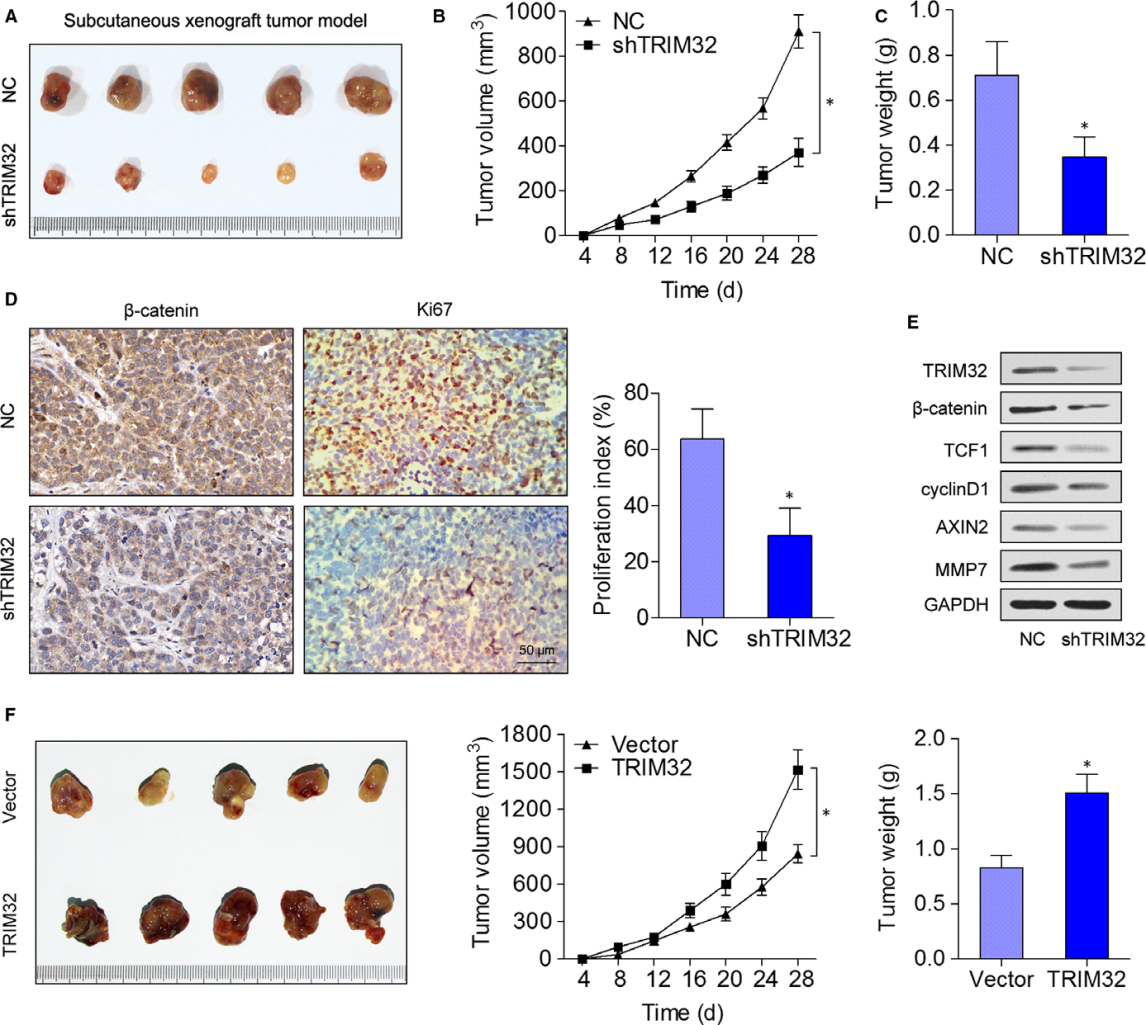
TRIM32 Promotes Gastric Tumour Growth in vivo. A, SGC7901 cells expressing shTRIM32 were subcutaneously injected into the nude mice, and the mice were monitored for 28 d. A representative picture of tumours thus formed is shown. B, A growth curve of tumour volumes was constructed every 4 d for 28 d. C, Tumour weight was measured. D, Tumour sections were subjected to haematoxylin and eosin (H&E) staining and IHC staining with an antibody against Ki‐67. E, Western blotting analyses of the expression of β‐catenin, TCF1 and cyclin D1 in tumour tissues. F, MKN28 cells were infected with the lentivirus expressing TRIM32 or the control lentivirus. The effect of TRIM32 overexpression on gastric cancer growth in nude mice. *P <* *0.05. Data represent mean ± SD

To verify the in vitro observations that the TRIM32 knockdown may have antitumour effects, a murine in vivo xenograft model of GC was established. SGC7901 cells infected with the shTRIM32 lentivirus (shTRIM32) or negative control (NC) cells were injected into the nude mice, and the tumour growth activity was measured. As compared to the control, the average tumour volume of the shTRIM32‐treated group was markedly reduced (Figure 5A,B). The average tumour weight was also significantly reduced in the shTRIM32‐treated group (Figure 5C). Accordingly, IHC staining showed that the levels of β‐catenin and Ki67 were consistent with the tumour volumes as well (Figure 5D). Western blotting revealed that the expression of β‐catenin, TCF1, cyclin D1, AXIN2 and MMP7 was remarkably reduced in shTRIM32‐inoculated tumour tissues (Figure 5E). On the other hand, lentiviral expression of TRIM32 resulted in accelerated xenograft tumour growth (Figure 5F). These data collectively indicated that TRIM32 functions as a novel tumour ‐enhancing molecule and positively regulates gastric tumour growth in vivo.

## 4 DISCUSSION

Although TRIM proteins have been studied in different types of cancer, and some members of the TRIM family have been found to be dysregulated and to participate in the pathogenesis of GC, the possible role of TRIM32 in GC is still unclear. In the present study, we demonstrated that TRIM32 is up‐regulated in GC tissues and cell lines compared with normal controls. The increase TRIM32 expression was probably related to the poor prognosis of GC, and this finding implied the possible clinical value of TRIM32 in GC. Besides, we showed that TRIM32 serves as an oncogene regulating GC cell proliferation, migration, and invasion by activating β‐catenin signalling.

Emerging data suggest that TRIM32 correlates with carcinogenesis. Liu et al^17^ reported that TRIM32 is frequently overexpressed in different types of human tumours, and TRIM32 overexpression promotes cellular oncogenic transformation and tumorigenesis in mice largely in a p53‐dependent manner. Cui et al^18^ found that up‐regulated TRIM32 correlates with enhanced cell proliferation and poor prognosis in hepatocellular carcinoma. Izumi et al^19^ reported that TRIM32 has the capacity to up‐regulate asymmetric cell division, which works against MYCN and can be considered a tumour suppressor in human neuroblastoma cells. Kano et al^20^ indicated that TRIM32 facilitates cell growth and migration via degradation of Abl interactor 2 in HEp2 cells. In our study, we found that TRIM32 is up‐regulated in GC tissues and cell lines, whereas increased TRIM32 expression correlated with a poor prognosis. These results are consistent with the findings of Ito and colleagues, in which up‐regulated TRIM32 was related to an increased risk of recurrence and an undesirable prognosis in patients with GC.^21^ Furthermore, a series of in vitro and in vivo experiments here revealed that a knockdown of TRIM32 suppresses cell proliferation, migration, invasion and tumour growth, whereas overexpression of TRIM32 yields the opposite results. Our study offers experimental evidence that TRIM32 is overexpressed in GC and promotes gastric tumorigenesis in vitro and in vivo.

β‐Catenin stabilization increases its nuclear protein levels, and constitutive activation of β‐catenin leads to tumorigenesis.^22^ Several lines of accumulating evidence point to the possible associations between the TRIM family and β‐catenin in cancers. For example, Wang et al^23^ found that TRIM29 expression correlates with elevated β‐catenin levels in pancreatic cancer, and β‐catenin function is required for TRIM29's oncogenic effects. Xue et al^24^ showed that TRIM33 as a tumour suppressor that can abrogate tumour cell proliferation and tumorigenesis by degrading nuclear β‐catenin. Knockdown of TRIM28 or TRIM44 suppresses the tumorigenesis by down‐regulating the Wnt/β‐catenin signalling pathway.^25^, ^26^ β‐Catenin can also be triggered by TRIM65 via enhancement of the ubiquitination of Axin1 in the progression of hepatocellular carcinoma.^27^ In the present study, we found that β‐catenin was down‐regulated by TRIM32 knockdown in SGC7901 and AGS cells and was up‐regulated in MKN28 cells with TRIM32 overexpression. In the nuclear fraction, β‐catenin expression was also remarkably enhanced in cells with TRIM32 overexpression and decreased in cells with silenced TRIM32. We confirmed that four representative downstream targets of β‐catenin (TCF1, cyclin D1, Axin2 and MMP7) are suppressed by TRIM32 shRNA and are enhanced by TRIM32 overexpression. In addition, when the β‐catenin‐specific inhibitor iCRT3 was added to the culture medium, up‐regulation of TRIM32 in MKN28 cells transfected with TRIM32 no longer enhanced the proliferation, migration and invasion of GC cells. These data suggest that TRIM32 exerts the tumour ‐promoting effect by activating the β‐catenin signalling pathway in GC.

In summary, our study suggests that TRIM32 plays a significant role in promoting cell proliferation, invasion and tumorigenesis thus bringing about an undesirable outcome among patients with GC by up‐regulating the β‐catenin signalling pathway. These findings may shed light on a new promising target for GC treatment.

## CONFLICT OF INTEREST

The authors declare that they have no competing interest.
